# Supplementary material for: Effect of immortal time bias on the association between immune-related adverse events and oncological outcomes following immune checkpoint inhibitors therapy for head and neck squamous cell carcinoma
Source: PLoS One. 2024 Nov 25;19(11):e0314209. doi: 10.1371/journal.pone.0314209 (PMC11588234; doi:10.1371/journal.pone.0314209)
Supplement: Attachment — Submitted filename: Reply to reviewers.docx [file pone.0314209.s003.docx]

**We would like to express our sincere gratitude for your thorough review and insightful comments on our manuscript. Your valuable feedback has greatly contributed to improving the quality of our work, and we have carefully considered all your suggestions in our revised submission. We truly appreciate the time and effort you have taken to review our manuscript.**

Reviewer #1: Tamura et al. investigated the association between immune-related adverse events (irAEs) and treatment outcomes in patients with head and neck squamous cell carcinoma (HNSCC) treated with immune checkpoint inhibitors (ICIs) and/or chemotherapies, focusing on the impact of immortal time bias. This retrospective study found significant differences in progression-free survival (PFS) and overall survival (OS) between patients with and without irAEs, demonstrating that irAEs might have a limited value as an independent prognostic factor for OS but not PFS when correcting for immortal time bias. The study concluded that the impact of irAEs on treatment outcomes was overestimated without considering immortal time bias and highlighted the need for clinicians to weigh the risks and benefits of ICI treatment carefully.

Despite its contributions, this manuscript has several shortcomings and weaknesses.

1) The novelty of this work is questioned, as previous studies have addressed immortal time bias in various cancer types. Although this study incorporates immortal time bias in its analysis, the findings do not significantly alter the previously understood associations in HNSCC and other cancers (Dall'Olio FG et al., 2021; Foster CC et al., 2021). It is essential to more clearly articulate the unique contributions and importance of this study.

**Response:**

**Reviewer 1 references a meta-analysis by Dall'Olio FG et al. (Immunotherapy, 2021). In their analysis, they note: “The extended Cox model with time-varying covariates is an alternative approach that considers a patient to be in the 'non-toxicity' group until they develop toxicity, at which point they shift into the toxicity group. This approach allows for the entire dataset to be used effectively. Unfortunately, none of the studies included in our analysis employed this more appropriate method.”**

**Additionally, the other cited article (Foster et al., Cancer, 2021) addressed immortal time bias using only landmark analyses. Therefore, we believe that our study, which utilizes an extended Cox model with irAEs as time-varying covariates, makes a meaningful contribution by applying a more appropriate and robust method to address immortal time bias.**

**We revised the introduction section as follows:**

**“Unfortunately, most previous studies did not consider immortal bias in their analyses or relied solely on landmark analyses, thus failing to adequately evaluate the association between irAEs and the therapeutic effect of ICIs ^4, 6^. Furthermore, these studies rarely incorporate extended Cox models with irAEs as time-varying covariates ^4-6^ .**

**This study aimed to investigate the true association between ICI outcomes and irAEs in patients with R/M HNSCC, addressing immortal time bias by utilizing both landmark analysis and an extended Cox model.”**

2) This study included a small sample size for the naïve analysis as its limitation. In addition, this study mentions the reduced sample size due to landmark analyses, which limits the statistical power of the findings. However, it does not provide a comprehensive assessment of how these limitations might impact the validity of the results. A more detailed discussion of the potential biases introduced by the small sample size and mitigation strategies would be necessary to strengthen the conclusions.

**Response:**

**In accordance with reviewer 1’s suggestion, we revised the discussion section as follows.**

**“	Second, it was a retrospective, single institutional study. Progression and irAEs were determined retrospectively using a chart review because of the lack of pre-specified criteria. To address the resulting bias, we conducted multivariable analyses and adjusted for confounding factors. Third, the number of patients was not large enough to provide sufficient statistical power. Landmark analyses further reduced the number of patients analyzed. Therefore, we could not conduct a multivariate analysis of response or disease control. Moreover, the small sample size led to increased variance and wide confidence intervals, making it difficult to achieve statistical significance. To overcome these limitations, a larger, prospective study—ideally multi-institutional—would be necessary.”**

3) While the authors have employed an extended Cox model to account for time-dependent variables, the manuscript does not fully explore how the timing of irAE onset may differentially impact patient outcomes. A more detailed analysis of the temporal aspects of irAE development, including the possible delayed effects on survival and progression, is recommended to add depth to the findings.

**Response:**

**We added additional analyses on early-onset irAEs and late-onset irAEs**

**“Patients with early-onset irAEs had a RR of 50.0%, while those with late-onset irAEs had a RR of 81.8%. Similarly, patients with early-onset irAEs had a DCR of 60.0%, compared to 90.9% for those with late-onset irAEs.”**

**“PFS was comparable between patients with early-onset and late-onset irAEs (p=0.159).”**

**“OS was comparable between patients with early-onset and late-onset irAEs (p=0.380).”**

“

4) The choice of using landmark analysis and EC models is appropriate, given the study’s objectives. However, the manuscript could benefit from a more explicit rationale for these choices compared to other possible methods, such as incorporating time-varying covariates in a traditional Cox model. In addition, the rationale for selecting specific landmark time points is not well-explained and requires further elaboration to enhance clarity.

**Response:**

**In accordance with reviewer 1’s suggestion, we revised the discussion section as follows:**

**“.Herein, we employed an EC model, which allows for the inclusion of onset timing as a time-dependent variable. In contrast, a traditional Cox model has a limitation in addressing the specific timing of irAE occurrence. “**

**We explored several landmark points for analyzing response rates and disease control rates (Table 2). Additionally, we have included Table S1 to present the survival analysis for different landmark points.**

5) The conclusion should clearly summarize the key findings and their implications without ambiguity. It should include a clear message of how immortal time bias affects the association between irAEs and oncologic outcomes, how clinicians should interpret this association to balance the risks and benefits of ICI therapy in clinical settings, and what alternative strategies could be considered.

**Response:**

**In accordance with reviewer 1’s suggestion, we revised the discussion section as follows:**

**“In conclusion, irAEs were identified as independent prognostic factors for OS but not for PFS. However, when immortal time bias is not accounted for, the association between irAEs and oncological outcomes in patients with HNSCC treated with ICIs tends to be overestimated. Our findings highlight the importance of accurately assessing the true prognostic impact of irAEs, as immortal time bias can exaggerate their perceived benefits. While irAEs may lead to life-threatening conditions, their occurrence offers limited predictive value for deciding whether to continue ICI treatment. Therefore, clinicians should not base decisions about ICI continuation on the presence of irAEs alone. Future research should explore more thoroughly the role of irAEs as potential on-treatment markers for ICI outcomes, ensuring that analyses rigorously account for immortal time bias to provide accurate and reliable conclusions.”**

6) The manuscript suggested further research to investigate the magnitude of irAEs associated with ICI outcomes. However, it would benefit from more specific recommendations for future studies, such as prospective designs, larger sample sizes, or the use of more comprehensive biomarkers.

**Response:**

**We revised the discussion section accordingly and added the following sentence:**

**“To overcome these limitations, a larger, prospective study—ideally multi-institutional—would be necessary.”**

7) This study, being retrospective and from a single institute, has inherent limitations. It is recommended to describe how the author attempted to overcome these limitations without validation cohorts.

**Response:**

**We revised the discussion section accordingly and added the following sentence:**

**“Second, it was a retrospective, single institutional study. Progression and irAEs were determined retrospectively using a chart review because of the lack of pre-specified criteria. To address the resulting bias, we conducted multivariable analyses and adjusted for confounding factors.”**

8) The study included 130 patients but analyzed 119 for an objective response. It should explain why 11 patients were excluded from the analysis. Also, Figures 1B and 2B included 108 patients for a survival analysis with a landmark analysis. Please clarify the patient number for each landmark analysis in Table 2.

**Response:**

**We revised the results section accordingly and added the following sentence:**

**“The remaining 11 patients were not evaluated for objective response due to death or deteriorated physical conditions.”**

**In accordance with reviewer 1’s suggestion, we added patient number in Table 2.**

9) In Table 1, 38 patients received the ICI therapy as 2nd or later lines. It is recommended to provide the details of previous treatment in these patients.

**Response:**

**In accordance with reviewer 1’s suggestion, we added the following in the result section;**

**“ICIs were administered as the first-line treatment for R/M disease in 70.8% of the patients. Most of the remaining patients received platinum-based chemotherapy in combination with cetuximab, either with docetaxel or fluorouracil, as prior therapy.”**

10) In Table 1, 38 patients received the ICI therapy with chemotherapy (fluorouracil + cisplatin). In these patients, it is recommended to describe how irAEs and AEs related to chemotherapy were differentiated and add the details of the irAEs and AEs.

**Response:**

**As outlined in the methods section, we classified AEs requiring corticosteroid treatment and endocrine AEs necessitating hormone replacement therapy as irAEs. Because other AEs may be immune-related or chemotherapy-related or related to other causes. Consequently, mild irAEs that resolved spontaneously may have been overlooked. Determining whether these mild AEs are immune-related is often challenging. Therefore, our definition of irAEs is more stringent than that used in previous studies.**

**We discussed this matter in the revised discussion section.**

**“First, we classified AEs requiring corticosteroid treatment and endocrine AEs necessitating hormone replacement therapy as irAEs. As a result, mild irAEs that resolve spontaneously may have been overlooked. This may have caused the low irAE incidence in our study. Furthermore, distinguishing whether these mild AEs are truly immune-related is often challenging, and accurately determining the timing of irAE onset can also be difficult. These factors may have influenced our findings.”**

11) Please update Table 1 to include all patient characteristics for both irAE (+) and irAE (-) groups, with the comparative statistics between the 2 groups.

**Response:**

**In accordance with reviewer 1’s suggestion, we added the comparative statistics in the revised table 1.**

12) irAEs occurred in 16.1% of patients, which is in the lower range compared to other ICI studies. Detailed information on any grade of irAEs or over grade 3 irAEs in each response criteria (CR, PR, SD, and PD), treatment for irAEs, and possible causes of low irAE incidence should be provided. Also, the association between the severe irAE (+) and oncologic outcomes should be added.

**Response:**

**As mentioned earlier, our definition of irAEs is more stringent, which may result in a lower reported incidence compared to previous studies. Due to this definition, our study primarily investigates the association between moderate to severe irAEs.**

13) In Table 2, the p-value in the 20-week landmark analysis for response is 0.064 with an asterisk (*), indicating statistical significance based on the legend of Table 2 (* p<0.05). It should explain why this is statistically significant, or if this is an error, please correct it.

**Response:**

**We corrected it accordingly.**

14) In Figures 1 and 2, it is recommended to add the p-value in each graph and the x-axis legend. Also, based on the median F/U period of 33.4 months, the authors should consider the extension of the x-axis.

**Response:**

**The median follow-up period for surviving patients was 33.4 months, while for all patients it was 13 months. Therefore, we reported the 1-year survival rate in the results section. Additionally, the number of patients at risk at 2 years in Figure 1 was only 15, so we believe extending the x-axis would not be necessary.**

**According to the reviewer’s suggestion, we added the p-value and the x-axis legend in the revised figures.**

15) In the Discussion section, the authors discussed predictive factors of ICI treatment, including PD-L1 expression. Basically, irAEs cannot be used to select patients or predict responses before starting treatment. This discussion is unfocused and irrelevant to their investigation. It is recommended to describe the relevant points for discussion.

**Response:**

**We appreciate your comment. In our discussion, we aimed to provide context for the role of irAEs as an on-treatment marker. To do so, we also discussed other on-treatment markers, such as imaging response, changes in LDH, and NLR. However, before addressing these, we felt it was important to briefly mention that existing predictive and prognostic biomarkers, such as PD-L1 expression, are limited in their utility before the initiation of treatment. This rationale was why we included a discussion on predictive and prognostic markers, to highlight the contrast with irAEs as an on-treatment marker.**

**We revised the discussion section focusing on the on-treatment markers.**

16) In Table 4, the correct terminology would be “multivariable analysis” rather than “multivariate analysis,” as this analysis focused on understanding how multiple predictors affect single outcomes and used Cox proportional hazard models to evaluate the influence of these variables on survival outcomes. If the authors want to keep the original terms, the relevant statistics and rationale should be provided.

**Response:**

**In accordance with the reviewer’s suggestion, we revised table 4.**

Reviewer #2: The present paper investigates the association between immune-related adverse events (irAEs) and survival in head and neck cancer patients treated with immune checkpoint inhibitors (ICIs), while accounting for immortal time bias. This topic is highly relevant, as previous studies have demonstrated this association but often did not adequately adjust for this important bias.
The study is well-conceived, with a robust statistical analysis and conclusions that are, overall, convincing. However, I have two comments for the authors.
First, I question the final sentence: “Therefore, the balance between the benefits and risks of ICI therapy must be carefully weighted in clinical settings.” The benefits and risks of ICI therapy are typically best summarized by findings from randomized controlled trials, not from exploratory studies like this one, which focus on predictors of efficacy.
The biological hypothesis suggesting that irAEs could serve as a surrogate marker in patients receiving immunotherapy is based on the idea that a stronger autoimmune response induced by ICIs may correlate with a more robust antitumor immune response. If irAEs are indeed part of the primary causal pathway in this context, their development could fully mediate the effect of ICIs on survival outcomes (Amoroso et al., ESMO Open, 2023). Accounting for immortal time bias may attenuate this association further at the individual patient level.

In my view, the primary conclusion of this study should emphasize that future research exploring the potential role of irAEs as surrogates for ICI efficacy must rigorously account for immortal time bias.

**Response:**

**In accordance with the reviewer’s suggestion, we revised the conclusion section.**

**“In conclusion, irAEs were identified as independent prognostic factors for OS but not for PFS. However, when immortal time bias is not accounted for, the association between irAEs and oncological outcomes in patients with HNSCC treated with ICIs tends to be overestimated. Our findings highlight the importance of accurately assessing the true prognostic impact of irAEs, as immortal time bias can exaggerate their perceived benefits. While irAEs may lead to life-threatening conditions, their occurrence offers limited predictive value for deciding whether to continue ICI treatment. Therefore, clinicians should not base decisions about ICI continuation on the presence of irAEs alone. Future research should explore more thoroughly the role of irAEs as potential on-treatment markers for ICI outcomes, ensuring that analyses rigorously account for immortal time bias to provide accurate and reliable conclusions.“**

Second, a potential limitation of the study is that the authors rely exclusively on clinician-reported adverse events (AEs) classified as irAEs. It is well established that patients often report adverse symptoms earlier and more frequently than clinicians. Furthermore, distinguishing irAEs from AEs arising from other causes, particularly in cases where ICIs are combined with other therapies, can be challenging. This introduces the possibility of misclassification bias, which may have influenced the study's findings.

**Response:**

**As reviewer 2 points out, it is difficult to accurately classify irAE and to estimate the timing of irAE occurrence. In accordance with the reviewer’s suggestion, we revised the discussion section.**

**“we classified AEs requiring corticosteroid treatment and endocrine AEs necessitating hormone replacement therapy as irAEs. As a result, mild irAEs that resolve spontaneously may have been overlooked. This may have caused the low irAE incidence in our study. Furthermore, distinguishing whether these mild AEs are truly immune-related is often challenging, and accurately determining the timing of irAE onset can also be difficult. These factors may have influenced our findings.”**
